# A Grander System of Human Life

**Published:** 1887-02

**Authors:** E. D. Babbitt

**Affiliations:** No. 20 University Place, N. Y.


### A GRANDER SYSTEM OF HUMAN LIFE,

INAUGURATED BY THE PACIFIC COLONY ON THE PRINCIPLE OF CO-OPERATION. HEALTH, HAPPINESS AND HARMONY POSSIBLE FOR EVERY MAN, WOMAN AND CHILD.

By E. D. BABBITT, M. D.

Many of my readers have doubtless heard of the great Colony which is forming with a view to settling on the Topolobampo Bay, Gulf of California, in the State of Sinaloa, Mexico. At the present writing, there are 4,700 persons connected with the corporation. Of these, 200 are already on the ground laying the foundation for Pacific City, and a large number more are getting ready to go within a few days. Every member takes one or more shares of stock at $10 each and subscribes to a system of rules and regulations, devised to a great extent by Mr. Albert K. Owen, Chief Engineer of the American and Mexican Pacific Railway Company. General Grant, Wendell Phillips, General Butler and other prominent persons were incorporators of this railroad, while ex-Secretary Windom is the president at this time. The Mexican Government has conceded large amounts of land to the railroad, the farther end of which at Topolobampo Bay constitutes the site of Pacific City. The Pacific Colony has already secured seventy to eighty thousand acres of this land, which for productiveness is unsurpassed in the world, while the climate is an everlasting spring and summer. The bay and harbor are among the most beautiful in the world, and possesses almost boundless quantities of oysters, turtles and fish of every kind. When the American and Mexican Pacific Railroad is built, Pacific City must naturally be a point of immense importance, as it will be about 800 miles nearer New York than San Francisco, for which reason the commerce of Asia, Australia and the Pacific Islands will naturally flow through it on its way to New York and Europe, instead of going through the more northern points. The Mexican Government has made excellent concessions to the colonists, and the Mexican people are receiving them with kindness and courtesy. Not far distant are the choicest woods, such as mahogany, ebony, etc.; in the mountains, through which the road is to pass, are the richest mines of gold, silver, coal, onyx, etc., while the most delicious tropical and other fruits are growing the year round. One fine crop a year can be produced without irrigation, but with irrigation three and in some cases four crops can be raised.

The air is remarkably pure and has not the damp and depressing character that it often has in our Southern States, or in our Northern States during summer. Malarial fevers, yellow fevers and sunstrokes are unknown there. Yellow fever has been known in Guaymas, a Mexican town on the gulf 200 miles farther north, but this is presumed to occur from the impure condition of the streets. What is singular about it, is that very few cases have been fatal. When cases of yellow fever were taken to Topolobampo Bay they began to get well immediately and did not prove contagious to those exposed to them. Lightning has never been known to strike people there, and blood diseases must be very easily managed in a climate which has almost a constant sunshine. From the first of June onward for about four months is what is called the rainy season, but even then there is generally a shower only once in three to five days. The temperature in the course of the year ranges at 52° to 86°, so it is neither so hot nor so cold as it is here. The nights are cool and pleasant. When the heat is at 86° there, it is said to be no more oppressive than it is here at 76°. This comes from the dryness and purity of the atmosphere.

The plan of the city as laid off by Mr. A. K. Owen is no doubt the finest that has ever been given to the world, having its park-like avenues from 100 to 200 feet broad, crossed by other streets at right angles, and these again by diagonal streets in both directions, while some thirty parks at equal distances are arranged for.

Mr. Owen has planned to have the streets run exactly east and west, north and south, etc., with the long side of the block, upon which the houses are to be built, running east and west. This is objectionable, for the north side of all houses which are in blocks must forever be in shade, and facts can be given to show that there are several times as much sickness and death on the shaded side of houses as upon the sunny side. Desiring that this shall be the model city of the world, especially as the plan is already so complete, I have written to him, begging him to wheel his plan around about 22½°, so that the north and south streets shall run that distance east of north and west of south. This will have two great advantages : first it will take the sunlight on all sides of a house every day, and secondly, the beds will catch the northward and some of the eastward electricities, or to be more explicit, the rooms will be so placed that the heads of the beds can come quite naturally to the east of north. The magnetic needle at Topolobampo points 10° east of the true north, showing that the strongest electrical or cooling forces move in that direction. Abundant facts could be given to show that these cooling forces should move toward the hottest part of the body, which is the brain, hence all sensitive persons sleep so much better with the head northward. But I have shown elsewhere that a milder grade of electricities move eastward, so that if a bed is turned with head east of north, it will catch the northern and some of the eastern forces, I have suggested also that the streets in one direction be called *avenues*, in another direction *streets*, in another *roads*, in another *places*, all streets, avenues, etc., to be named from numbers, such as 1st st., 2nd st., etc., 1st ave., 2nd ave., etc. To give the streets of a town arbitrary names, such as Locust street, or Walnut Street, is to burden people with names that have no significance as streets. No person in a lifetime of residence in Philadelphia or Washington, systematic as those cities are, will be able to tell you the names and locations of all the streets, although only a part of them are arbitrary; but with a scientific system, founded on numerals, the names and the very location of all the streets in a city can be learned in two minutes. Carry out this idea and then count each block 100, on the Philadelphia plan, and what an amount of time and blundering will be saved. I say thus much on this subject because new towns and cities are being constantly laid out all over the country, where this folly of arbitrary names is ever being repeated, and endless trouble made for all the future residents of a town. Burden a town at the start with false methods and for thousands of years to come the people will have to suffer for it.

The Pacific Colony then having as they do a grand location, with a climate unsurpassed in the world, with the finest salt mine in the world on their own property, with unlimited treasures of the sea and neighboring mountains, with mountains of porphyny on their very city site, with the ability to raise three crops a year on their farming land, by aid of irrigation, and with a prospective commerce that bids fair to be very extensive, are placed under very happy auspices.

But I come now to a feature of this colony which is of greater importance than any of the foregoing advantages, namely the principle of co-operation in harmony with which they act. These people go forward not only to secure happy homes for themselves, but to work out a great principle in the social and civil upbuilding of man. Seeing the heinousness of the present money-grabbing and brutal system in which the strong and the cunning trample under foot the weak and the helpless, in which the rich often grow so rich that they do not know what to do with their money, while millions grow so poor that they do not know what to do without money, they have laid out a system of real equality and harmonial liberty, far transcending the equality and liberty of the freest republics on earth. While a large number of them are children of industry and plain laboring people, a goodly number will be people of decided ability and fine accomplishments. Mr. Albert K. Owen, the projector and the chairman of the colony, is a man of cosmopolitan character, and has laid out a beautiful and broad scheme of integral co-operation, undoubtedly the most practical thing of the kind that has thus far been given to the world. His work, consisting of 208 pages, called *Integral Co-operation*, explains his theory in full, and is devoted to the constitution, conditions and surroundings of the Pacific Colony. All interested in these great social movements should send thirty cents to the John Lovell Publishing Co., 14 Vesey street, New York, for this work. Mr. John Lovell, himself an accomplished and upright business man, is the treasurer of the Pacific Colony, and the other directors are highly able and intellegent persons. Ex-Governor Rice, Jesse Grant, son of Gen. Grant, and many other well-known people are stock-holders in the company.

Mr. Owen discovered that Topolobampo Bay was an unsurpassed point for a great city and for a grand community. An ordinary greedy capitalist, whose god is Mammon, seeing such a point and realizing its future growth, would have bought up all the finest land in its vicinity and then, as it began to develop into a city, would charge from five to five hundred times as much as it cost him, thus skinning the poor unfortunate people who should be seeking for homes or places of business in the vicinity. This is a feature of the competitive system. Now note the difference between that and a righteous system. Mr. Owen discovers this great opportunity for gaining choice land, and, aided by the other directors, buys it up, not for his own aggrandizement, but for the whole people. The whole people hold it for themselves, and the whole people, including women as well as men, elect those to manage their business whom they deem the wisest. At a small price a certan amount of land is signed over to each of the colonists, and at cost price homes are built for them and paid for out of their wages. The colonists will have their commissariat and their bazaar, where provisions and goods of all kinds will be furnished at wholesale prices. Schools, lectures, nurseries and libraries will be furnished free to all. If any one is sick a nurse is provided, and insurance arrangements are made so that in old age one can rest from labor and have a certain fund provided. Those having infants and very small children can have them cared for in the nursery or the kindergarten school more skillfully than it could be done at home. Arrangements for cooking, laundry work, etc., will be made by the wholesole, and the drudgery and unscientific character of ordinary housework will be avoided. No such words as master and servant will be tolerated, for all are masters and all servants, each one receiving employment from the directors which he or she has helped to elect. The organization will not be so base as to force woman to work for less pay than a man would receive for the same amount and character of work. Eight hours a day will be considered sufficient for a man to labor, perhaps still less time for a woman. Taxation of all kinds will be done away with, under their system, and the whole body will be like one family, the interest of one being the interest of all.

No private speculation will be allowed. Although every one will have his own house and ground sacred to himself, it can never be sold outside of the company, else the old Sodom of competition will be introduced.

They are arranging to have some beautiful oriental and other styles of architecture for their homes. I have suggested a plan of houses which may come under the head of *solar architecture*, for the great sun-healing movement which is now commencing is destined to revolutionize the homes of the whole people. One style suitable for all sun-lands, such as Mexico, California, Colorado and some adjacent States, as well as Southern France, Oriental Asia, etc., would be the flat roofed structures with an ornamnetal balustrade around the whole top, at least four and a half feet high. This enclosurre forms a *solarium* for nude sun bathing, where members of the family, in imitation of the ancient Romans, can walk in nature's undress style at mid-day, quite unobserved of the outside world, and drink in life and power from the sunlight, and oxygen from the breezes. But we can have something more scientific than the Roman solaria. In the middle of the balustrade, on the south side of the house, I would have a pavillion, in the front of which I would place panes of blue, red, amber and purple glass. This would give a fine effect to the house while the sun bather could move his settee and lie down under the color which his system most requires. For rousing and warming the blood he would use the red ; for animating the nerves he would use the amber ; for quieting excitable blood and nerves he would use the blue, etc. The breeze that sweeps over the top of a house in the evening of a hot day is like heaven, and if a lamp should be hung in the pavillion the colors would show like a fairy palace. Another form of solar architecture would be to have a steep Gothic style of roof formed much like a letter A, cut off half way down where the crossing line comes. The balustrade and pavilion could be built on this narrower top to form the solarium, while the inclosed lower part would constitute a *solar story*, the windows lying flat with the roof so as to let the sun far into the room even when it is high in the sky. The rooms of a solar story would be superb for a solar sweat-bath, called the thermolume. If people could realize that sunless rooms become filled with bacteria and other impurities, and are unwholesome, they would see the importance of the arrangement spoken of above.

In cold climates, where snow sometimes collects in large masses in winter, the roof can be carried all the way up, the lower part of the A

constituting the solar story, while part of the upper portion of the roof can be made of glass which can slide open or remain closed, according to the weather. Families thus provided with a solarium and a thermolume would gain a wonderful power and generally escape all sickness.

It is usually supposed, that warm, southern climates tend to weaken people, and under our false system of excessive dressing this is true. For our athletes we go to our Northern States and to Canada, rather than to our Southern States. To give power, it requires electricity on the one hand, combined with a due amount of heat and light on the other. Electricity abounds in all cold conditions, or, to be more accurate, coldness is but a coarser form of electricity. Is it probable then, that these colonists going to the warmer climate of Mexico, will lose theit vital energy and become debilitated? This is not necessary at all if the people but understand themselves, for the wonderful sunlight of that region contains a mightier and more refined grade of electricity than the force know as *cold*. I have ascertained that the blue, indigo and violet rays of sunlight constitute a form of electricity, just as the red, orange and yellow constitute a grade of heat. I have demonstrated in the last June number of HALL'S JOURNAL OF HEALTH that the races who go nude in the sunlight and air are at least twice as powerful as the nations that wear clothing, and the following passage from Mr. James W. Steele's "Mexico by Palace Car" will show that some of the humbler classes of Mexicans who wear but little clothing, and frequently go with legs and feet bare to the sun must be twice as strong as ourselves.

"The peasant's gait is quick and all his movements active. He is a notoriously fast walker. Slight in figure and thick set, he will and often does carry a burden of 300 pounds, and go off with it at a jog trot. * * Three men and sometimes two will carry a piano a dozen squares."

It is plain then that if these colonists, when they reach the sunny realm of Sinaloa where their home is to be, will have their solaria for solarizing their bare bodies, or will remove a part of their clothing while working in the field, they will gain a greater physical power and keenness of intellect than American or Englishmen at their own homes possess.

It will be seen that this harmonious system of things will do away with the necessity of the dreadful and nerve-rasping battles of life which our present crude, social and civil conditions necessitate. Physicians know very well that disease and prostration, as well as inebriety, are often caused by the pressure of poverty and the unfairness of those with whom one has to deal. They know too, that under a happy condition, more beautiful and harmonious children will be born, and a nobler manhood and womanhood developed. If this article were not already too long, I could show from statistics that the laborer does not get one-fifth of what he produces in the actual value and rise in property, and I could show too, that when this co-operative plan is carried out by the government in the management of railroads for the people, the cost of travel will not be one fiftieth of what it is at the present time. Why not have government control railroads and telegraphs as well as post-offices?

I have communicated these facts to HALL'S JOURNAL OF HEALTH because under the new management it is presenting new and grander truths for the up-building of the suffering world. The press of New York and other cities will give large space to show the poverty and misery of the people, but their superficial souls will not allow a single column to show how to do away with this poverty and misery, although we give abundant facts to prove our points.

*No.* 20 *University Place, N. Y.*

